# Eye-Tracking Evidence for TACOM-Based Assessment of Procedural Task Complexity in a Nuclear Power Plant Full-Scope Simulator

**DOI:** 10.3390/s26175487

**Published:** 2026-08-29

**Authors:** Huan Xiao, Pengcheng Li, Wenming Chen, Jiayuan He, Zetian Tao

**Affiliations:** 1 School of Resources Environment and Safety Engineering, University of South China, Hengyang 421001, China; xiaohuan9599@163.com; 2 Graduate School, University of South China, Hengyang 421002, China; 3 School of Nuclear Science and Technology, University of South China, Hengyang 421001, China

**Keywords:** nuclear power plant, task complexity, TACOM, eye tracking, partial least-squares regression, human factors engineering

## Abstract

Emergency and operating procedures in nuclear power plants usually require operators to search for information, judge system states, and make control decisions across several linked interfaces. Conventional TACOM assessment quantifies the structural complexity of such procedure-guided tasks, but it does not directly show how operators visually process the task during execution. This study examined whether eye-tracking features can provide preliminary process-level evidence for TACOM-based assessment, rather than replace the TACOM framework. We extracted 25 eye-tracking features from 21 nuclear engineering graduate students while they completed 17 SGTR/SLOCA procedure fragments in an M310 full-scope simulator. A partial least-squares regression model predicted task-level TACOM scores, with RMSE = 0.357, MAE = 0.300, and R^2^ = 0.538 under leave-one-task-out cross-validation. The RMSE corresponded to 18.3% of the observed TACOM range (2.070–4.017), and prediction was more strongly associated with observed task ranking (Spearman’s rho = 0.775) than with exact linear calibration (Pearson’s r = 0.750). Feature analyses suggested that fixation–duration variability, fixation dwell, pupil response, gaze dynamics, and spatial sampling jointly carried TACOM-related information. Exploratory subdimension analyses further indicated that task scope was most consistently associated with fixation dwell and fixation-time proportion, whereas task uncertainty was more closely associated with pupil variability and spatial entropy. These findings suggest that eye tracking may complement TACOM by describing execution-process demands, although the evidence remains correlational and limited by the small task set, graduate student sample, and task interface variability. Future studies should validate the signatures with licensed operators, larger multi-scenario task sets, independent TACOM scoring, and step-level AOI analyses.

## 1. Introduction

During abnormal and accident management in nuclear power plants, operators do not make decisions in an unconstrained environment. They work through emergency or abnormal operating procedures that require information confirmation, state diagnosis, and control actions [1,2,3]. These procedural tasks are basic units of human reliability in the main control room. Missed actions, delayed judgement, or deviations from the prescribed sequence can weaken accident mitigation [3]. Task complexity is therefore a central performance-shaping factor in operator performance, procedure design, crew training, and human–system interface evaluation [4,5,6]. The issue has become more prominent in digital main control rooms, where alarms, parameter trends, soft controls, and display pages are more tightly coupled. A complex procedure can force operators to search across interfaces, integrate dispersed cues, and maintain several logical conditions before acting.

To quantify this form of procedural complexity, Park and Jung proposed the task complexity measure (TACOM) [7,8]. TACOM describes procedural tasks through five aspects: the amount of information to be processed, the number of actions, the logical relations among steps, the system knowledge required, and the difficulty of establishing judgement criteria. These submeasures are further integrated into composite dimensions, such as task scope, task structurability, and task uncertainty. Compared with simple step counts or single expert ratings, TACOM converts the information, action, and judgement requirements embedded in procedure text into comparable task-level scores [9,10]. Subsequent work has supported the validity of TACOM from several perspectives, including its association with task completion time, workload ratings, unsafe actions, and performance time in digital main control rooms [3,5,7,9,10,11].

Most existing TACOM validation, however, still relies on outcome measures such as execution time, subjective workload, or unsafe behaviors. These measures are valuable because they show whether complex tasks have performance consequences, but they are usually observed after task execution. They offer less direct evidence about how operators allocate attention, search interface information, and process judgement conditions during the task itself. In other words, TACOM has helped answer which procedural tasks are structurally more complex and whether complex tasks are associated with poorer performance. It remains less clear whether this structural complexity leaves observable traces in process-level behavior. For procedure assessment in nuclear power plants, this question is practical: if complexity scores can be linked to execution behavior, TACOM can serve as a static structural index and as one layer in a behavior-based evidence chain for procedure optimization and training evaluation.

Eye-tracking measures are theoretically relevant to TACOM because they describe how operators allocate visual attention while procedural information is being perceived, interpreted, and integrated. In situation awareness terms, procedure execution requires the perception of indicators, the comprehension of plant state, and the projection of likely consequences [12]. In visual search terms, operators must select and revisit task-relevant display regions under changing information demands [13]. Fixation duration and fixation variability are commonly treated as indicators of visual dwell and local information processing [14], whereas pupil diameter and pupil variability are sensitive to processing effort and arousal [15]. Gaze dispersion, spatial entropy, and transition measures describe the breadth and organization of screen space sampling [16,17,18,19,20]. These measures are not equivalent to TACOM, which is a structural score, but they can test whether tasks with larger information scope, weaker structurability, or higher uncertainty evoke different execution process signatures.

On this basis, the present study positions the eye-tracking model as a behavioral validation and exploratory supplement to TACOM rather than as a replacement for it. Using an M310 pressurized water reactor full-scope simulator, we recorded eye movements while participants completed 17 emergency procedure fragments with different TACOM scores. We then examined whether task-level eye-tracking features could predict TACOM scores, which feature groups contributed to prediction, and whether the eye-tracking signatures differed across TACOM subdimensions.

The study makes three contributions. First, it shifts the question from simply predicting TACOM scores to testing whether procedural structural complexity has an observable eye-movement trace during task execution. Second, it evaluates PLSR performance against a mean baseline and reports duration-control, feature-group, and uncertainty analyses to avoid overinterpreting the small task-level sample. Third, it explores whether TACOM subdimensions show different eye-tracking signatures, while treating these patterns as descriptive evidence that requires independent validation.

## 2. Materials and Methods

### 2.1. Experimental Platform

**Figure 1:**
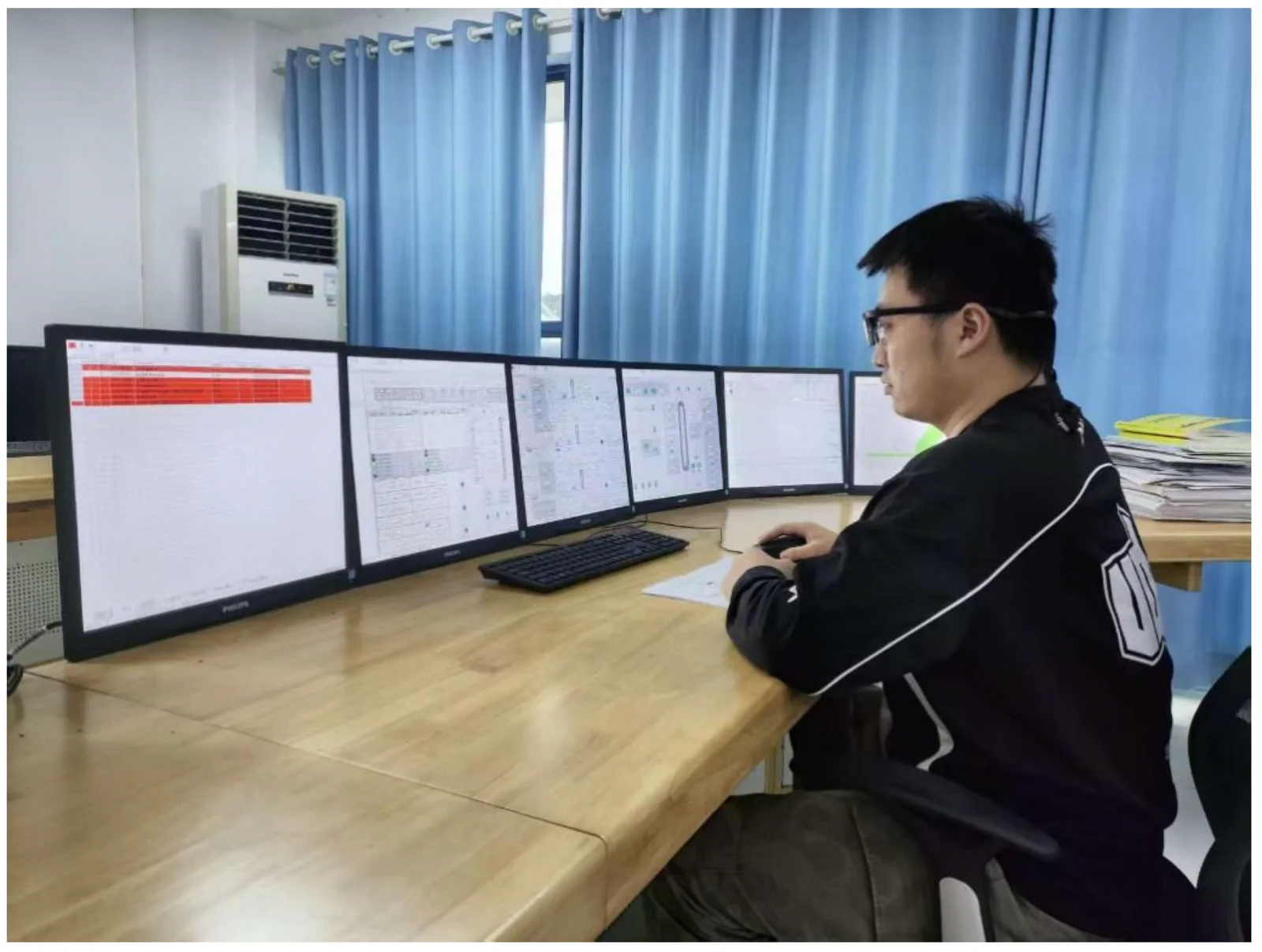
M310 pressurized water reactor full-scope simulator used in the experiment.

The experiment was conducted on an M310 pressurized water reactor full-scope simulator at the Human Factors Engineering Research Institute of the University of South China (Figure 1). The simulator comprises six 19-inch display screens and presents the plant-state parameters, alarms, procedure pages, and control interfaces needed for SGTR and SLOCA emergency operations. Eye movements were recorded with Tobii Pro Glasses 2 (Tobii AB, Stockholm, Sweden) at a sampling rate of 100 Hz. All participants used the same simulator workstation and task list; however, different procedure fragments can activate different display pages and navigation patterns. Therefore, spatial gaze variables are interpreted as screen space behavioral summaries rather than pure measures of structural complexity, and this interface factor is revisited in the limitations.

### 2.2. Participants

Twenty-one participants were recruited for the experiment. All were graduate students in nuclear-related disciplines and had at least one year of nuclear engineering education. The participants had normal or corrected-to-normal vision and were proficient computer users. This cohort was suitable for controlled method development because participants had similar domain education and received the same pre-experiment training, but it does not represent licensed nuclear operator crews. Accordingly, all generalizations to operational crews are framed as preliminary and require future validation with licensed or mixed-expertise samples.

### 2.3. Procedural Tasks

Seventeen typical emergency procedure fragments were selected from SGTR and SLOCA scenarios for complete information flow calculation and experimental execution. The fragments were derived from actual emergency operating procedures of a nuclear power plant (Appendix A Table A1) and covered parameter confirmation, system-state judgement, and control actions. Tasks were selected according to three criteria: they could be completed independently in the simulator environment; they involved different numbers of information elements and information-processing processes; and they showed clear differences in TACOM scores. Each participant completed all 17 tasks. The TACOM score for each procedural task was calculated before the experiment to keep task design and quantitative assessment consistent.

### 2.4. Experimental Procedure

The experiment followed a standardized procedure. First, all participants completed approximately 30 min of training to become familiar with the procedural tasks, simulator operation, and experimental instructions. Tobii Pro Glasses 2 calibration was then performed for each participant before formal recording. During the formal experiment, each participant received a task objective, executed the corresponding procedure fragment on the simulator, and completed all 17 tasks in a randomized order. The same briefing, task instructions, and rest schedule were used across participants. Participants rested for 2–3 min after every two tasks and for 20 min between the SGTR and SLOCA accident scenarios. The total experiment lasted approximately 90–120 min per participant.

### 2.5. Eye-Tracking Feature Extraction

For readability, Table 1 summarizes the functional feature groups, and the complete variable dictionary is provided in Appendix B. Feature extraction covered recording duration, fixation, saccade, pupil, gaze spatial distribution, gaze dynamics, and fixation–transition features. The exported participant–task records included gaze coordinates, pupil diameter, and sample-level event labels. Fixation and saccade features were computed from detected events, pupil features were computed from valid pupil samples, and spatial variables were computed in screen pixel coordinates. Rate and proportion variables were normalized by valid recording duration, and spatial entropy was computed over a 4 × 3 grid covering the display space. Invalid or eyes-not-found samples were used for data quality checking rather than interpreted as task complexity signals. The participant–task features were averaged to the task level before modeling because TACOM labels describe tasks rather than individual trials.

### 2.6. TACOM Measurement

We used the task complexity measure (TACOM) to quantify the structural complexity of each procedural task. TACOM was originally developed for evaluating task complexity in emergency operating procedures of nuclear power plants. Its central premise is that task complexity depends on the number of procedure steps, the amount of information that must be processed, the logic and number of required actions, the system knowledge that must be invoked, and the cognitive resources needed to establish judgement criteria [7,9]. Human factors studies using full-scope simulators have further shown that TACOM scores are positively associated with unsafe behaviors or human error occurrences, indicating that the measure captures complexity factors relevant to procedural execution [3].

Following the TACOM framework, each task was first coded using five submeasures. Step information complexity (SIC) represents the amount of information that operators must obtain, identify, and process. Step logic complexity (SLC) represents the logical relations and action order complexity among procedure steps. Step size complexity (SSC) represents the number of actions required to complete the task. Abstraction hierarchy complexity (AHC) represents the level of system knowledge and functional understanding needed to execute the task. Engineering decision complexity (EDC) represents the difficulty of establishing judgement criteria and making engineering decisions when the task condition is uncertain.

When a task consisted of several consecutive procedure steps, this study used the task rather than the individual step as the TACOM calculation unit. Task complexity is not a simple sum of step complexity. Different steps can share the same state information, equipment objects, or judgement conditions, and direct summation would double-count part of the complexity. After task boundaries were defined, the information, actions, logical relations, and knowledge requirements within each task were coded as a whole, and a task-level TACOM score was then calculated.

TACOM scores were assigned before eye-tracking model fitting and were based on procedure content and TACOM coding rules, not on gaze features or prediction outcomes. This ordering reduced direct leakage from eye-tracking data into the response variable. Nevertheless, because the labels were produced within the research team rather than by an independent blinded operator panel with reported inter-rater reliability, potential scoring subjectivity remains a limitation.

After the five submeasures were scored, they were integrated into three composite dimensions using previously established hierarchical weights: task scope (TS), task structurability (TR), and task uncertainty (TU):

(1) TS=0.716×SIC+0.284×SSC

(2) TR=0.891×SLC+0.109×AHC

(3) TU=EDC

The final TACOM score was calculated using a weighted Euclidean norm:

(4) $$TACOM=\sqrt{\left(0.621\times {TS}^{2}+0.239\times {TR}^{2}+0.140\times {TU}^{2}\right)}$$

Here, TS reflects the scope of information and actions covered by the task, TR reflects internal step relations and dependence on system knowledge, and TU reflects uncertainty in judgement criteria during execution. A higher TACOM score indicates greater integrated demand on information processing, action organization, and decision judgement. TACOM scores were calculated for all experimental tasks and used as continuous complexity targets in the eye-tracking models.

### 2.7. PLSR Modeling

Let X denote the standardized task-level eye-tracking feature matrix and y denote the vector of continuous TACOM scores. PLSR decomposes X into latent score vectors T while maximising the covariance between these latent variables and y [17,18]. The basic decomposition is:

(5) $$X=TP^{T}+E$$

(6) y=Tq+f

where P is the loading matrix for X, q is the response loading vector, and E and f are the residual terms for the feature matrix and response variable, respectively. After the latent variables have been obtained, the model can be written as:

(7) $$y=XB+b_{0}$$

where β is the regression coefficient vector transformed back from the latent-variable space, and β0 is the intercept. Unlike principal component regression, which focuses only on variance within X, PLSR gives priority to eye-tracking feature combinations that are relevant to the prediction target. It is therefore suitable for the present modeling setting, where the number of task samples is small and the predictors are strongly correlated [19].

In this study, the PLSR input consisted of 25 eye-tracking variables and the output was a single continuous TACOM score. During model training, X was first standardized to zero mean and unit variance within the training data, and the same transformation was then applied to the held-out task in each validation fold. We also fitted feature-group models and a duration-control model that removed duration_s to examine whether prediction was driven mainly by task duration. Because eye-tracking variables are strongly correlated, PLSR coefficients were used only as descriptive multivariate contributions and not as directional or causal effects.

Model performance was evaluated with continuous prediction metrics, including root mean square error (RMSE), mean absolute error (MAE), coefficient of determination (R2), Pearson’s correlation, and Spearman’s rank correlation. RMSE was interpreted relative to both the mean-score baseline and the observed TACOM range. Spearman’s correlation was reported because a practically relevant use of the model is ranking procedure fragments by relative complexity, whereas Pearson’s correlation and RMSE reflect exact linear calibration and absolute score error.

## 3. Results

We first treated TACOM as a task-level structural complexity target and tested whether eye movements during procedural execution contained complexity information that generalized to unseen tasks. All prediction analyses were performed at the task level. The 21 participants completed 17 procedural tasks, yielding 357 participant–task observations, which were then aggregated into 17 task-level eye-tracking feature vectors. Each task was represented by 25 eye-tracking features.

### 3.1. Distribution of TACOM Scores Across Procedural Tasks

**Figure 2:**
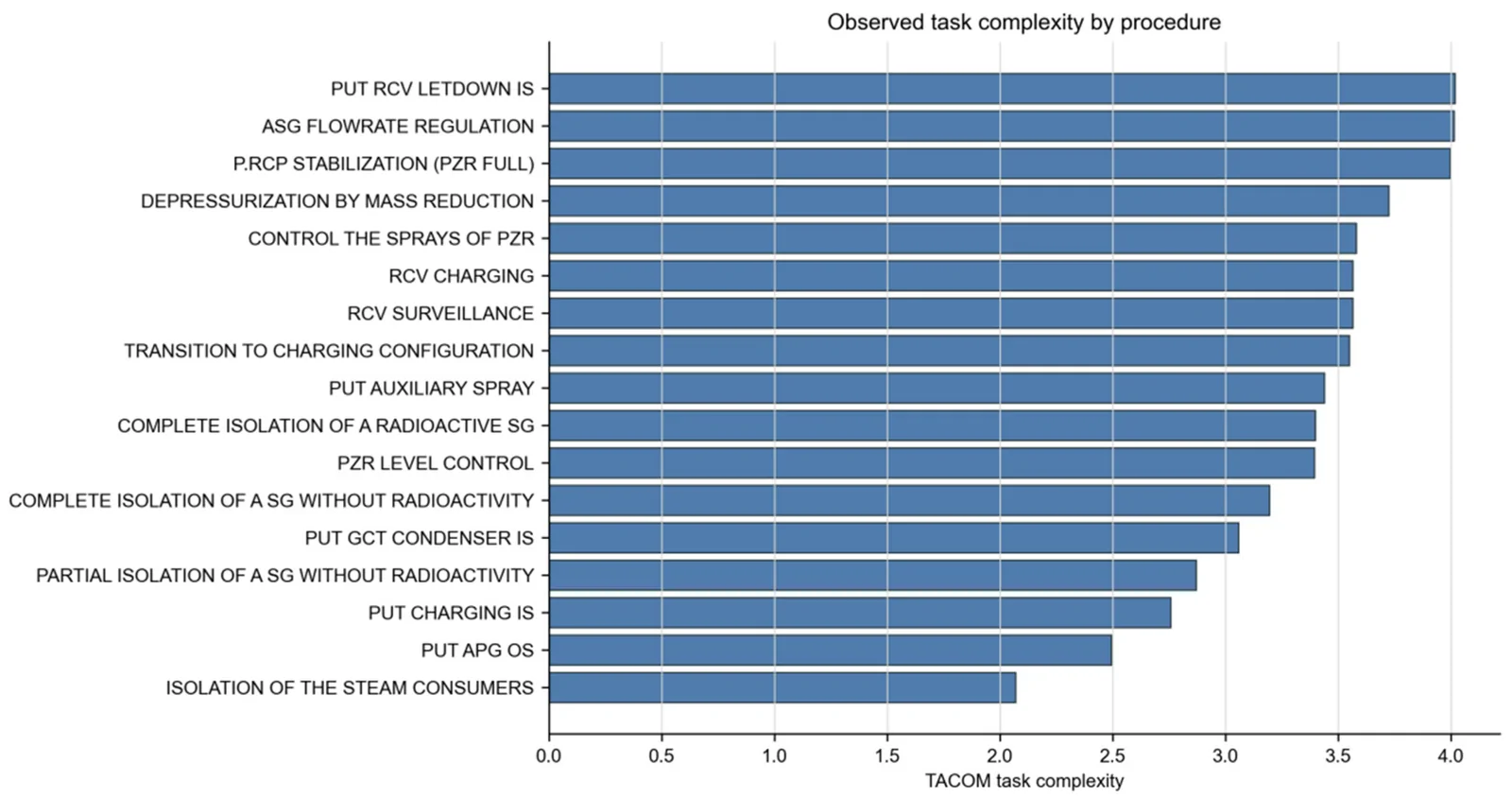
Distribution of continuous TACOM scores across the 17 procedural tasks.

As shown in Figure 2, TACOM scores for the 17 procedural tasks ranged from 2.070 to 4.017, with a mean of 3.335 and a standard deviation of 0.542. The selected SGTR and SLOCA procedure fragments therefore covered low, medium, and high levels of complexity. The tasks with the highest TACOM scores were PUT RCV LETDOWN IS, ASG FLOWRATE REGULATION, and P.RCP STABILIZATION (PZR FULL). The tasks with the lowest scores were ISOLATION OF THE STEAM CONSUMERS and PUT APG OS. The continuous score gradient shown in Figure 2 indicates that the prediction target was not a simple high–low classification, but a task-level complexity continuum suitable for regression modeling.

### 3.2. Task-Level TACOM Prediction Using Eye-Tracking Features

**Figure 3:**
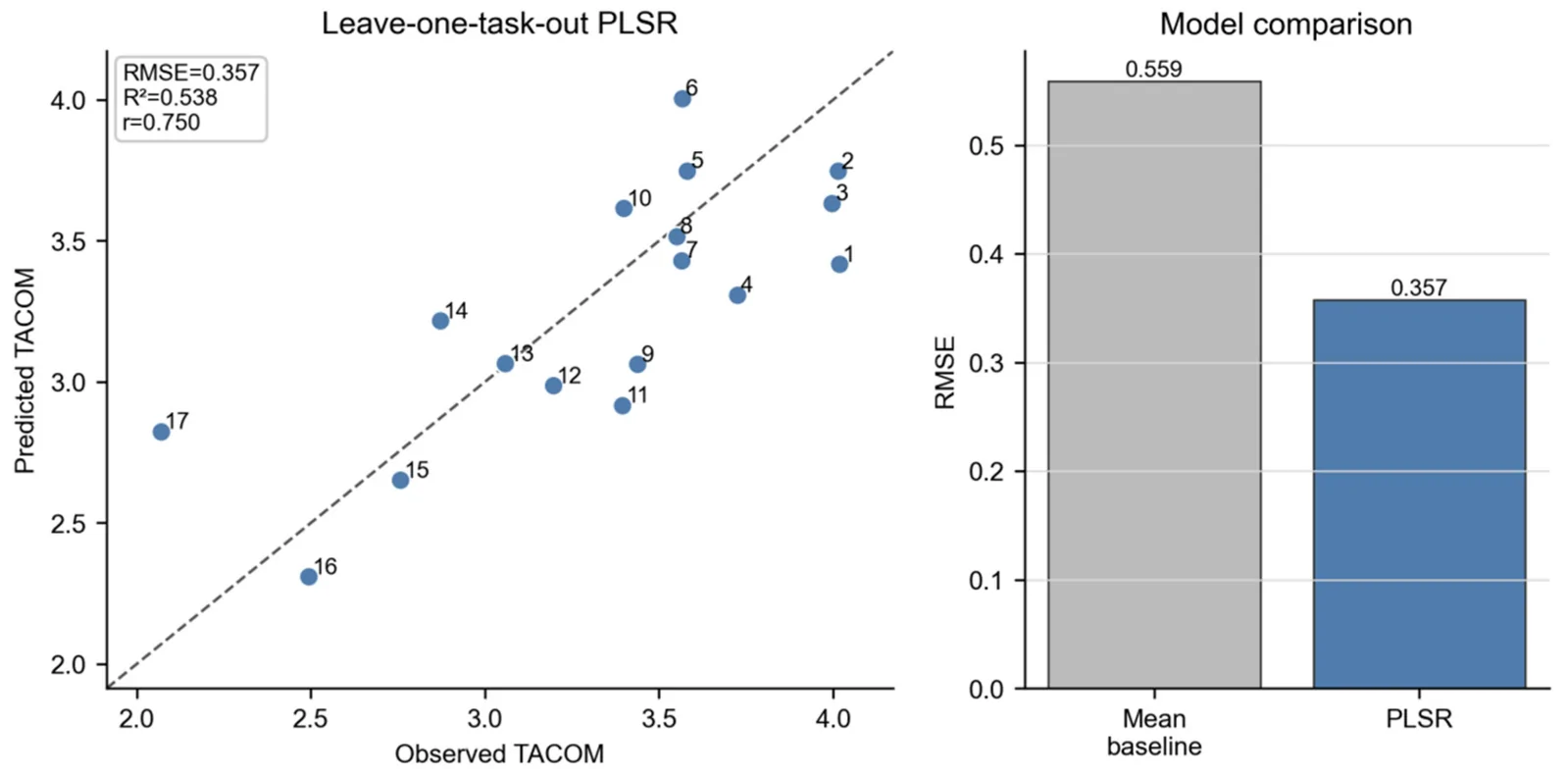
Continuous TACOM prediction performance of the PLSR model.

As shown in Table 2 and Figure 3, the PLSR model achieved RMSE = 0.357, MAE = 0.300, R^2^ = 0.538, Pearson’s r = 0.750, and Spearman’s rho = 0.775 under leave-one-task-out cross-validation. The RMSE was lower than the mean baseline RMSE of 0.559 and corresponded to 18.3% of the observed TACOM range (2.070–4.017). The model therefore captured a moderate amount of task-level variance but should not be interpreted as a precise score replacement tool. The stronger Spearman correlation compared to Pearson correlation indicates that the model preserved relative task ordering slightly better than exact linear calibration, which may reflect the small task set, limited score range, local nonlinearities, or sensitivity to individual task residuals.

The positive association between predicted and observed TACOM scores in Figure 3 further shows that the model did not simply regress to the mean. Instead, it preserved a substantial part of the ordering among procedural tasks. As an auxiliary check, continuous predictions were discretised using the same TACOM thresholds. The median-split classification produced an accuracy of 0.765 and a macro F1 score of 0.757; the three-level classification produced an accuracy of 0.706 and a macro F1 score of 0.676. These classification results are reported only to support the rank preservation interpretation, whereas the main analysis remains the continuous prediction of TACOM.

### 3.3. Predictive Contribution of Eye-Tracking Feature Groups

To examine whether PLSR prediction depended on a single eye-tracking dimension, we performed feature-group ablation analyses. As shown in Table 3, each single feature group performed worse than the full feature set. Using fixation features alone yielded RMSE = 0.500 and R^2^ = 0.095; using pupil features alone yielded RMSE = 0.485 and R^2^ = 0.149; and using spatial and gaze dynamics features alone yielded RMSE = 0.481 and R^2^ = 0.165. Saccade features alone performed below the mean baseline (R^2^ = −0.398).

The ablation results in Table 3 suggest that TACOM-related execution behavior was distributed across fixation stability, pupil variation, spatial search, and gaze dynamics rather than being carried by one isolated feature family. Importantly, the model without duration_s achieved RMSE = 0.313 and R2 = 0.646, indicating that the predictive signal did not depend on raw task duration and that duration_s may have added noise in this task set. This result is treated as duration control evidence only: task duration remains conceptually entangled with procedural complexity, and future analyses should use step-normalized or AOI-normalized gaze metrics to isolate complexity-specific behavior more directly.

### 3.4. Eye-Tracking Indicators Associated with TACOM Scores

**Figure 4:**
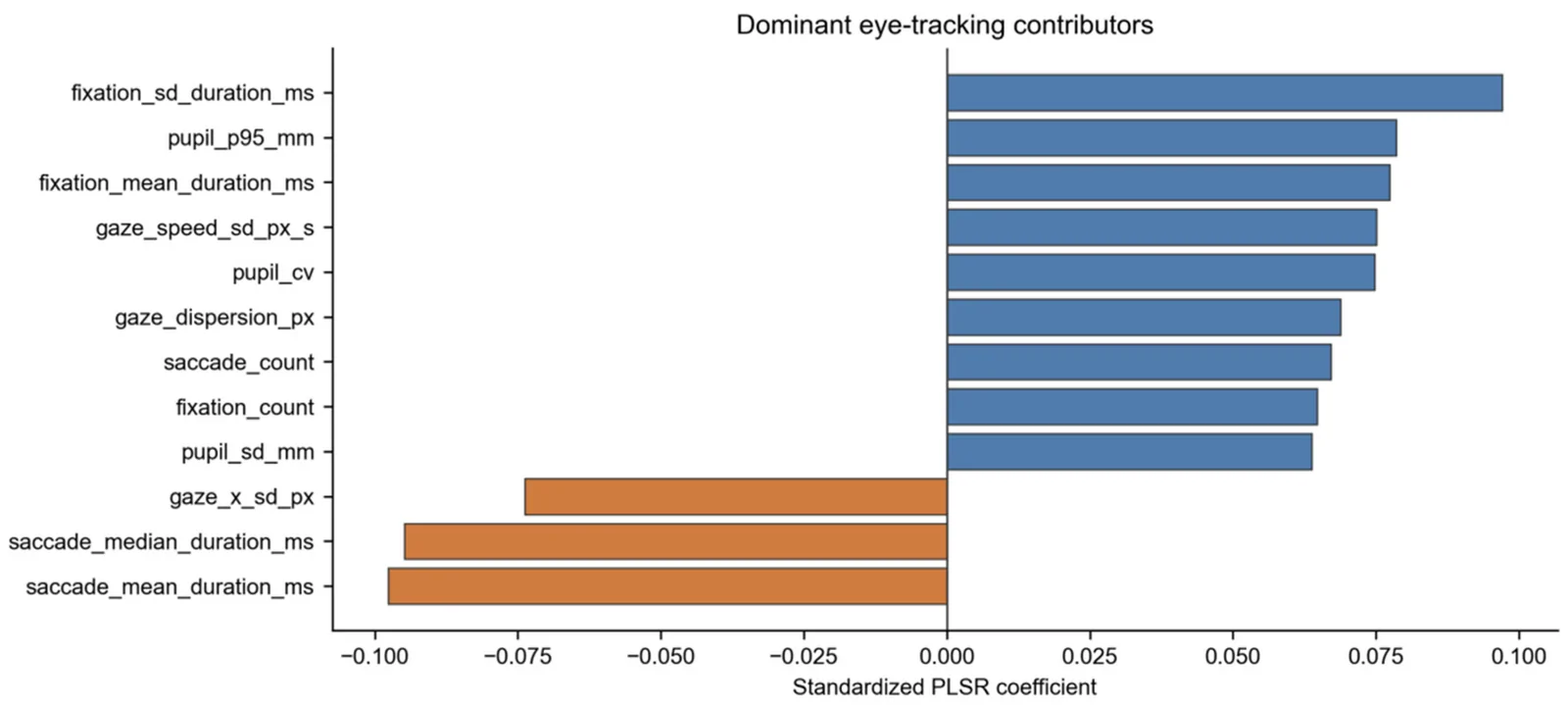
Ranking of eye-tracking feature contributions in the PLSR model.

To interpret the behavioral information used by PLSR, we examined standardized coefficients and task-level univariate correlations. Because many eye-tracking features are mutually correlated and PLSR forms latent components from shared variance, coefficient signs should not be read as independent causal effects. Positive or negative coefficients indicate how each standardized feature contributed within this fitted multivariate combination conditional on the other features. As shown in Table 4 and Figure 4, variables with large absolute coefficients therefore identify candidate behavioral signatures rather than isolated mechanisms.

The univariate correlations provide a complementary interpretation. Table 4 shows that fixation_sd_duration_ms was positively associated with TACOM (Pearson’s r = 0.642, Spearman’s rho = 0.718), and pupil_p95_mm was also positively associated with TACOM (Pearson’s r = 0.610). These correlations are consistent with the PLSR pattern and suggest that high-complexity procedure fragments tended to involve more variable fixation dwell and stronger pupil response. However, because correlations were estimated from 17 task-level observations, they should be interpreted as descriptive associations that guide subsequent confirmatory analysis.

### 3.5. Exploratory TACOM Subdimension Signatures

To address whether eye-tracking signals mapped differently onto TACOM components, we conducted an exploratory task-level subdimension analysis. Because the TACOM dimensions were themselves correlated (TS-TACOM r = 0.992, TU-TACOM r = 0.927, and TR-TACOM r = 0.362), these results were treated as descriptive signatures rather than independent confirmatory tests. Task scope showed the clearest fixation-related pattern, with positive associations for fixation_median_duration_ms (r = 0.659, 95% CI 0.319 to 0.845), fixation_total_prop (r = 0.642, 95% CI 0.425 to 0.849), and pupil_p95_mm (r = 0.619, 95% CI 0.171 to 0.854). Task uncertainty showed a related but distinct pattern involving pupil_cv (r = 0.593, 95% CI 0.319 to 0.801), spatial_entropy_4×3 (r = 0.566, 95% CI 0.056 to 0.840), and fixation_transition_mean_px (r = 0.568, 95% CI −0.030 to 0.851). In contrast, step-logic complexity and task structurability were less consistently reflected in global eye-tracking summaries: SLC was associated with lower gaze_speed_sd_px_s (r = −0.490, 95% CI −0.719 to −0.224), while TR showed a weaker association with fixation_rate_per_min (r = −0.415, 95% CI −0.797 to 0.179). These patterns suggest that task scope and uncertainty may be more visible in whole-task eye-tracking summaries than logic relationship components, which may require step-level AOI or sequence analyses.

### 3.6. Uncertainty and Task-Level Error Analysis

Because the task-level sample contained only 17 observations, we used 5000 task-level bootstrap resamples to estimate uncertainty in the PLSR results. The bootstrap interval should be interpreted as uncertainty within this limited task set rather than as evidence of generalization to new plants, interfaces, or accident scenarios. This uncertainty is important because small task sets can make latent-variable estimates unstable and can increase the risk of overfitting.

A permutation test that fixed the predictions and permuted the TACOM labels showed that the observed task–label correspondence was better than random assignment. The permutation result supports a non-random association between predicted and observed task complexity, but it does not establish causal mechanisms or guarantee that the same coefficients would hold in another scenario set.

**Figure 5:**
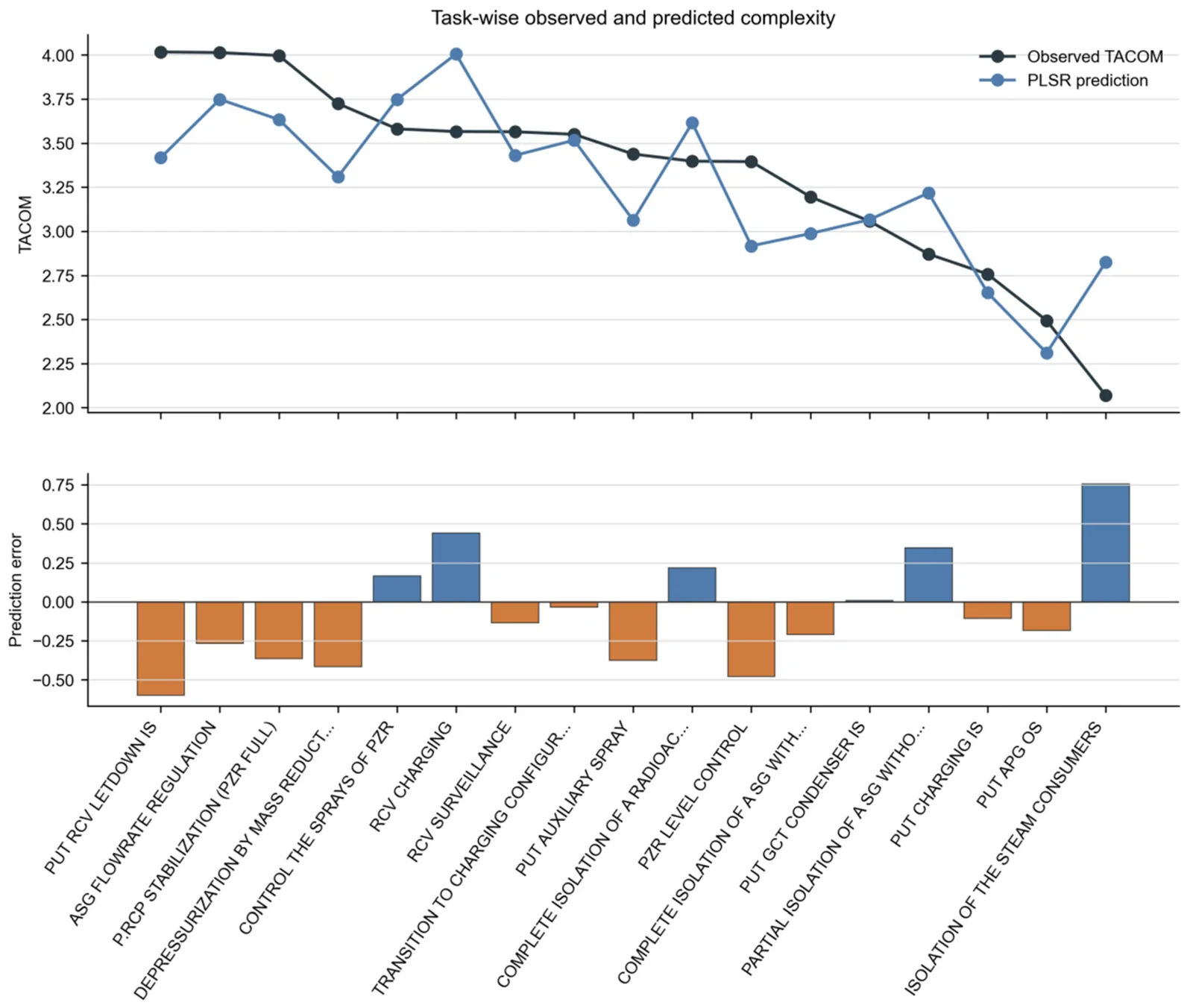
Task-level prediction errors.

**Figure 6:**
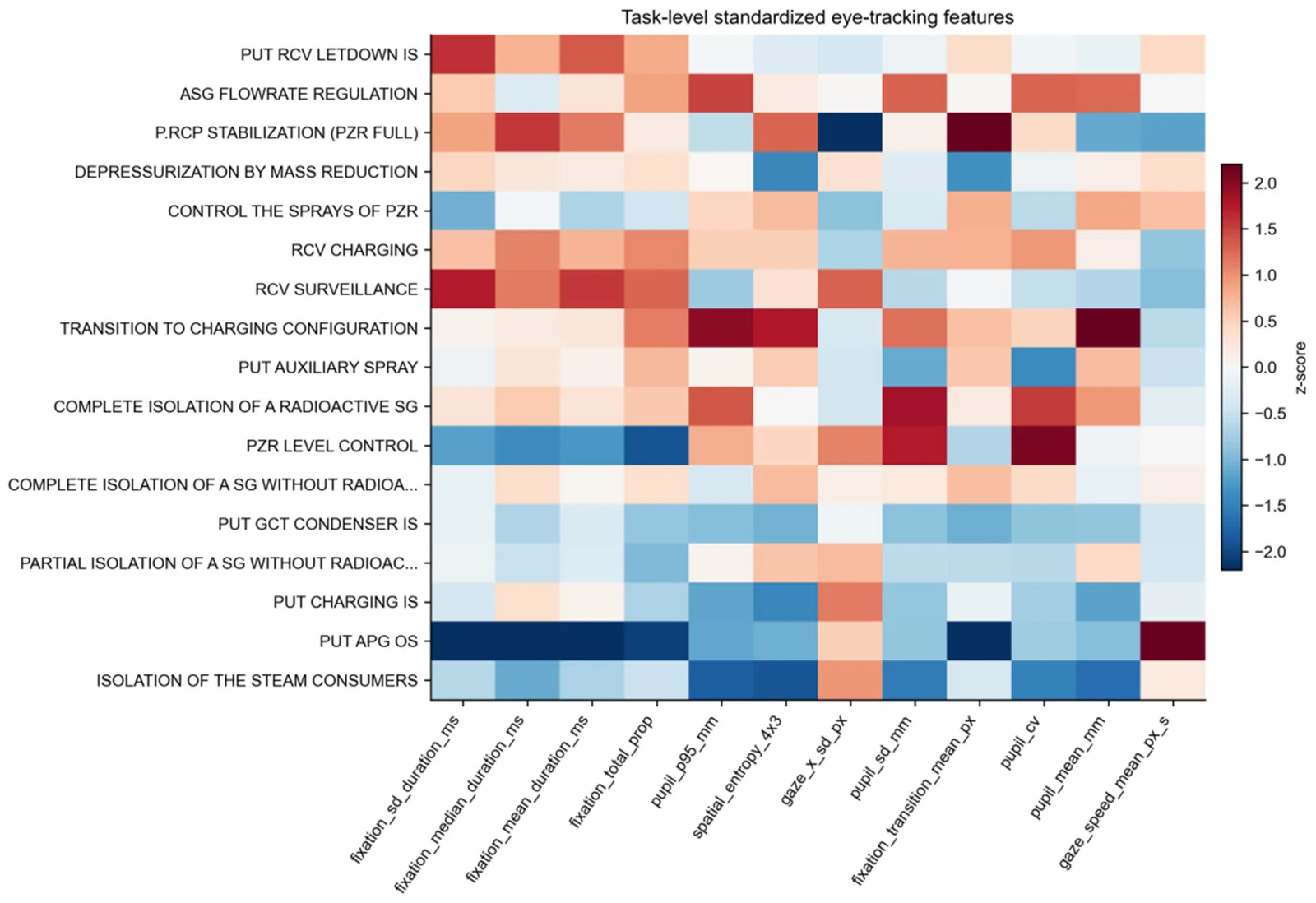
Task-level heatmap of standardized eye-tracking features.

Task-level residuals further defined the boundary of the method (Figure 5 and Figure 6 and Table 5). PUT GCT CONDENSER IS had an error close to zero, and TRANSITION TO CHARGING CONFIGURATION also showed good agreement between observed and predicted scores. By contrast, ISOLATION OF THE STEAM CONSUMERS was substantially overestimated, whereas PUT RCV LETDOWN IS and PZR LEVEL CONTROL were underestimated. These boundary cases suggest that some procedural complexity may arise mainly from logical judgement, system consequences, or step-to-step dependencies that are not fully captured by global task-level eye-tracking statistics.

**Figure 7:**
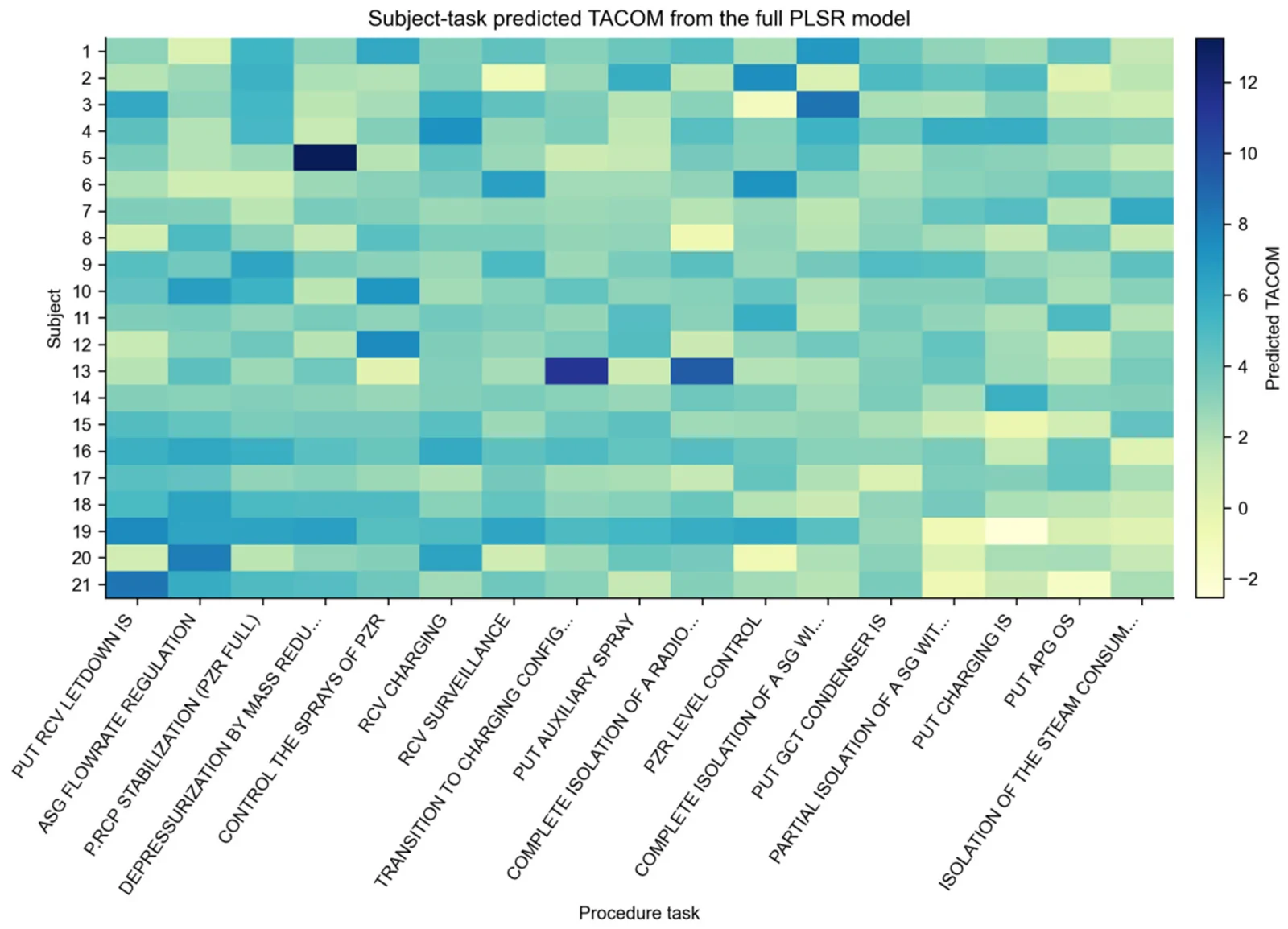
Participant–task heatmap of PLSR-predicted complexity.

Figure 7 extends the analysis to the participant–task level and shows individual differences in predicted complexity across tasks. At the present stage, the PLSR output is best viewed as exploratory process-level evidence for identifying tasks or participant–task executions that merit closer review. It should not be interpreted as an operator ability measure or as a standalone operational assessment.

## 4. Discussion

The central finding of this study is not that PLSR is already a sufficiently robust complexity prediction model. Rather, the results suggest that procedural structural complexity leaves a measurable, but preliminary, behavioral trace in eye movements during task execution. The model outperformed the mean baseline, preserved much of the relative ranking of TACOM scores, and retained above-baseline performance when duration_s was removed. These findings support the idea that eye tracking can add execution process evidence to TACOM, while the small number of tasks and correlational design require cautious interpretation.

The pattern is consistent with the theoretical composition of TACOM and with broader human factors accounts of operator cognition. TACOM describes information amount, action scope, step logic, system knowledge, and engineering judgement. In situation awareness terms, these demands require operators to perceive relevant indicators, comprehend their relation to plant state, and anticipate consequences [21]. In visual search terms, higher task scope and uncertainty should alter fixation dwell, revisits, and spatial sampling across displays [22,23]. The observed fixation, pupil, and entropy patterns are therefore theoretically plausible, although they remain indirect behavioral markers rather than direct measurements of structural complexity.

The feature-group ablation results further support the multidimensional nature of the behavioral representation. Models based only on fixation, pupil, saccade, or spatial and dynamic features were weaker than the full-feature model, indicating that TACOM-related information was distributed across several aspects of gaze behavior. The improved performance after removing duration_s further suggests that the signal was not merely a proxy for longer task completion time. Nevertheless, duration is closely related to procedural execution, and future studies should combine time-normalized, step-normalized, and AOI-normalized indicators to separate structural complexity from execution pace.

This work also complements recent attempts to estimate nuclear power plant task complexity using physiological signals. Electroencephalographic and deep learning approaches can extract rich cognitive information, whereas eye tracking provides a more transparent description of how operators sample and revisit interface information. The present results indicate that fixation dwell, pupil response, gaze dynamics, and spatial entropy may help explain why some TACOM dimensions are more observable during task execution than others [24]. This role is supplementary: eye tracking can help prioritize tasks for further analysis, but it cannot by itself validate task complexity, workload, or human error probability.

The residual pattern offers a useful diagnostic view of the method’s boundary. For some tasks, predicted values were close to TACOM scores, suggesting that structural complexity was reflected in global eye movement summaries. For other tasks, prediction errors were larger, which may occur when complexity is driven by procedure logic, system-state interpretation, or display page navigation that is not captured by whole-task statistics. Simulator interface variability is particularly relevant here: different procedure fragments can require different pages, controls, and display regions, so gaze dispersion may partly reflect screen design rather than task complexity. AOI-based analyses and layout controls are therefore necessary before stronger claims can be made.

## 5. Limitations and Future Work

Several limitations should be acknowledged. First, the effective modeling unit consisted of only 17 tasks. Leave-one-task-out cross-validation, bootstrap uncertainty estimation, and permutation testing reduce but do not remove this limitation. The small task set can make PLSR latent variables unstable and increases the risk that apparent feature contributions are scenario-specific. Future work should expand the task set across additional accident scenarios and procedure fragments before the model is used for general prediction.

Second, participants were nuclear-related graduate students rather than licensed operators. The student sample helped produce a controlled experimental process, but students differ from licensed operators in procedural fluency, diagnostic strategies, interface familiarity, and plant operation experience. Therefore, the present signatures should not be generalized directly to operational crews. Future validation should include licensed operators or mixed-expertise cohorts to test whether the same eye-tracking signatures hold under realistic operating expertise.

Third, this study used task-level aggregated eye-tracking features and did not fully exploit the temporal structure within procedure steps. The logical relations, engineering judgement requirements, and interface navigation patterns embedded in a procedure may appear only at specific decision points. In addition, different simulator pages and display layouts may influence spatial gaze dispersion independently of TACOM. Future work should combine procedure–step alignment, AOI definitions, transition sequences, simulator logs, and display layout controls to determine whether gaze dispersion reflects structural complexity or interface design.

Fourth, TACOM scores are derived from procedure–structure assessment and should not be treated as real-time mental workload or human error probability. The present TACOM labels were assigned before model fitting, but they were not produced by an independent blinded operator panel with reported inter-rater reliability. Future work should include independent TACOM coders, inter-rater agreement checks, and sensitivity analyses such as permutation importance or SHAP-style interpretation. Thus, this study provides preliminary behavioral validation evidence, not proof that eye tracking can replace TACOM or independently determine task complexity.

## 6. Conclusions

This study examined the behavioral observability of TACOM-based procedural complexity assessment. Using data from 21 participants, 17 SGTR/SLOCA procedural tasks, and 25 eye-tracking features, PLSR predicted task-level TACOM scores with RMSE = 0.357, MAE = 0.300, and R2 = 0.538. Predicted and observed scores were positively associated (Pearson’s r = 0.750; Spearman’s rho = 0.775), and the model outperformed the mean score baseline. Feature and subdimension analyses suggested that fixation variability, fixation dwell, pupil response, gaze dynamics, and spatial sampling carried TACOM-related information, especially for task scope and uncertainty.

Overall, the evidence supports a cautious and supplementary role for eye-tracking data in TACOM-based procedure assessment. Eye tracking may help reveal how structural task demands are expressed during execution and may help identify tasks that require closer procedure, interface, or training review. However, the conclusions remain limited by the correlational design, small task set, graduate student sample, researcher-generated TACOM labels, and absence of step-level AOI controls. Larger studies with licensed operators, independent TACOM scoring, richer task scenarios, and interface-controlled gaze analysis are needed before operational deployment.

## Figures and Tables

**Table 1:** Functional groups of eye-tracking variables used for PLSR modeling. Full variable definitions are provided in Appendix B.

Feature Group	Variables	Interpretive Role in This Study
Time baseline	duration_s	Valid recording duration; used as a control variable and tested in the duration exclusion analysis.
Fixation	fixation count/rate, mean/median/SD duration, fixation-time proportion	Visual dwell, fixation stability, and the proportion of task time devoted to local information processing.
Saccade	saccade count/rate, mean/median/SD duration, saccade-time proportion	Gaze shift frequency and visual transition behavior during procedural execution.
Pupil	mean diameter, SD, coefficient of variation, 95th percentile	Effort- and arousal-related physiological variation during task execution.
Spatial distribution	horizontal/vertical gaze SD, gaze dispersion, 4 × 3 spatial entropy	Breadth and distribution of screen space visual sampling.
Gaze dynamics and transitions	path per second, mean/SD gaze speed, mean fixation-transition distance	Movement variability and transitions between information sources.

**Table 2:** Continuous prediction performance under task-level leave-one-task-out cross-validation.

Model	RMSE	MAE	R^2^	Pearson’s r	Spearman’s rho
PLSR	0.357	0.300	0.538	0.750	0.775
Mean baseline	0.559	0.445	−0.129	NA	NA

**Table 3:** Feature-group ablation results for PLSR.

Feature Group	No. of Features	RMSE	MAE	R^2^	Pearson’s r	Description
All 25 features	25	0.357	0.300	0.538	0.750	Full eye-tracking feature set
Fixation only	7	0.500	0.390	0.095	0.441	Fixation frequency, duration, and stability
Pupil only	4	0.485	0.385	0.149	0.518	Pupil dilation and variability
Spatial/gaze dynamics only	8	0.481	0.376	0.165	0.451	Gaze dispersion, speed, entropy, and transitions
Without duration_s	24	0.313	0.250	0.646	0.807	Control model excluding valid recording duration
Saccade only	6	0.622	0.531	−0.398	−0.096	Saccade count and saccade duration

**Table 4:** Eye-tracking features with large PLSR coefficients and their task-level correlations with TACOM.

Feature	Standardized PLSR Coefficient	Pearson’s r	Spearman’s rho
saccade_mean_duration_ms	−0.098	−0.215	−0.353
fixation_sd_duration_ms	0.097	0.642	0.718
saccade_median_duration_ms	−0.095	−0.344	−0.484
pupil_p95_mm	0.079	0.610	0.478
fixation_mean_duration_ms	0.077	0.641	0.706
gaze_speed_sd_px_s	0.075	0.094	0.199
pupil_cv	0.075	0.519	0.493
gaze_x_sd_px	−0.074	−0.549	−0.549
gaze_dispersion_px	0.069	0.212	0.304
saccade_count	0.067	0.018	0.110

**Table 5:** TACOM scores, PLSR predictions, and prediction errors for the 17 procedural tasks.

Procedural Task	TACOM	Predicted TACOM	Error	Observed Rank	Predicted Rank
PUT RCV LETDOWN IS	4.017	3.418	−0.599	1	8
ASG FLOWRATE REGULATION	4.014	3.748	−0.265	2	3
P.RCP STABILIZATION (PZR FULL)	3.997	3.634	−0.363	3	4
DEPRESSURIZATION BY MASS REDUCTION	3.724	3.309	−0.416	4	9
CONTROL THE SPRAYS OF PZR	3.581	3.749	0.167	5	2
RCV CHARGING	3.566	4.005	0.440	6	1
RCV SURVEILLANCE	3.564	3.431	−0.134	7	7
TRANSITION TO CHARGING CONFIGURATION	3.550	3.517	−0.033	8	6
PUT AUXILIARY SPRAY	3.438	3.062	−0.375	9	12
COMPLETE ISOLATION OF A RADIOACTIVE SG	3.398	3.615	0.218	10	5
PZR LEVEL CONTROL	3.395	2.917	−0.478	11	14
COMPLETE ISOLATION OF A SG WITHOUT RADIOACTIVITY	3.196	2.987	−0.208	12	13
PUT GCT CONDENSER IS	3.057	3.066	0.009	13	11
PARTIAL ISOLATION OF A SG WITHOUT RADIOACTIVITY	2.871	3.218	0.347	14	10
PUT CHARGING IS	2.757	2.651	−0.106	15	16
PUT APG OS	2.494	2.310	−0.184	16	17
ISOLATION OF THE STEAM CONSUMERS	2.070	2.825	0.755	17	15

## Data Availability

The original contributions presented in this study are included in the article. Further inquiries can be directed to the corresponding author.

## Appendix A

**Table A1:** The shortened emergency shutdown procedure used in this study.

Operation Procedure	Details of Step and If-Yes Response	If-No Response
1. RCV SURVEILLANCE	1.1 At least one RCV pump IS?	Go to 1.15
	1.2 RCV letdown flowrate > 5 m^3^/h?	Go to step 1.5
	1.3 T° downstream regenerative heat exchanger letdown side > 190 °C?	Go to 1.5
	1.4 Put RCV letdown OS	
	1.5 RCV 227VP closed?	Go to 1.7
	1.6 Return	
	1.7 RCV Charging flowrate > 9 m^3^/h	Go to 1.14
	1.8 Reduce the auxiliary spray flowrate < 9 m^3^/h	
	1.9 RCV Charging flowrate > 9 m^3^/h?	Go to 1.14
	1.10 RCV letdown flowrate > 5 m^3^/h?	Go to 1.13
	1.11 Open RCV 050VP	
	1.12 Return	
	1.13 Close RCV 227 VP	
	1.14 Return	
	1.15 Implement RCR sheet N° 19 (Putting IS charging pumps)	
	1.16 Go to 1.2	
2. ASG FLOWRATE REGULATION	2.1 P.SG1 > 7 Bar.g	Go to 2.12
	2.2 Put TAFP ASG 003PO IS	
	2.3 ASG 003PO IS?	Go to 2.10
	2.4 Put ASG 001PO and 002PO OS	
	2.5 Regulate ASG 013VD according to ASG flowrate requested	
	2.6 ASG flowrate regulable?	Go to 2.8
	2.7 Return	
	2.8 Ask to implement RFLL sheet N° LL110 (Regulating ASG 013VD)	
	2.9 Return	
	2.10 Put TAFP ASG 004PO IS	
	2.11 ASG 004PO OS?	Go to 2.4
	2.12 SG Level NR < −1.8 m?	Go to 2.18
	2.13 Put ASG 001PO and 002PO IS	
	2.14 Augment SG level by ASG 012VD > −0.58 m NR	
	2.15 ASG flowrate regulable?	Go to 2.17
	2.16 Return	
	2.17 Ask to implement RFLL sheet N° LL111 (Regulating ASG 012VD)	
	2.18 SG Level NR < 0.9 m?	Go to 2.20
	2.19 Go to 2.14	
	2.20 Close ASG 012VD	
	2.21 ASG flowrate of this SG = 0 m^3^/h?	Go to 2.23
	2.22 Return	
	2.23 Put ASG 001PO and 002PO OS	
	2.24 Ask to implement RFLL sheet N° LL112 (Closing ASG 012VD)	
	2.25 Return	
3. PUT GCT CONDENSER IS	3.1 At least one signal P12: RPA 039KS or RPB 039KS present?	Go to 3.5
	3.2 Set GCT 503KC on P MODE	
	3.3 Unlock GCT condenser by GCT 501KC and 502KC	
	3.4 GCT 503 and 504KS lit?	Go to 3.7
	3.5 Set GCT 401KU on MANU	
	3.6 Regulate the cooling by GCT 401KU	
	3.7 Return	
4. PUT RCV LETDOWN IS	4.1 Open RCV 010VP	
	4.2 Open RCV 003VP	
	4.3 Open RCV 002VP	
	4.4 RCV 010VP open?	Go to 4.19
	4.5 RCV 003VP open?	Go to 4.19
	4.6 RCV 002VP open?	Go to 4.19
	4.7 Primary monophase RCV 409KC on RCP 037MP?	Go to 4.21
	4.8 Set RCV 013VP on MANU at 0%	
	4.9 Open the three RCV letdown orifices	
	4.10 Regulate RCV 013VP at the value of reactor coolant pressure attained	
	4.11 Set RCV 013VP on AUTO	
	4.12 RCV Letdown < 5 m^3^/h?	Go to 4.15
	4.13 Put letdown OS	
	4.14 Return	
	4.15 Close RCV 250VP	
	4.16 Close RCV 257VP	
	4.17 Close RCV 258VP	
	4.18 Return	
	4.19 Ask to implement RFLL sheet N° LL136 (Line-up letdown)	
	4.20 Return	
	4.21 Set RCV 013VP at 50% on MANU	
	4.22 Open one RCV letdown orifice	
	4.23 Regulate the letdown pressure to 25 bar.g by RCV 013VP	
	4.24 Set RCV 013VP on AUTO	
	4.25 If letdown flowrate < 5 m^3^/h, regulate the number of orifices to obtain the flowrate > 5 m^3^/h	
	4.26 Go to 4.12	
5. CONTROL THE SPRAYS OF PZR	5.1 Confirm RCP 016KG on MANU	
	5.2 Close RCP 001VP	
	5.3 Close RCP 002VP	
	5.4 Can not read the position of RCP 001VP and 002VP in KIC?	Go to 5.7
	5.5 ΔT sat > 20 °C?	Go to 5.19
	5.6 Return	
	5.7 RCP 001VP close?	Go to 5.19
	5.8 RCP 002VP close?	Go to 5.19
	5.9 ECP1 in progress?	Go to 5.24
	5.10 Set RCP 401KU on MANU and resume the signal at 0%	
	5.11 Set RCP 001VP on AUTO	
	5.12 Set RCP 002VP on AUTO	
	5.13 RCP 001VP close?	Go to 5.16
	5.14 RCP 002VP close?	Go to 5.16
	5.15 Return	
	5.16 Close RCP 001VP	
	5.17 Close RCP 002VP	
	5.18 Return	
	5.19 Put RCP 001PO OS	
	5.20 Put RCP 002PO OS	
	5.21 ECP1 in progress?	Go to 5.24
	5.22 Fallback mode: NS/RRA	
	5.23 Launch fallback time: Immediate	
	5.24 Return	
6.DEPRESSURIZATION BY MASS REDUCTION	6.1 Put the heaters OS	
	6.2 Regulate RCPp inj. flowrate to 3 × 1.4 m^3^/h/pump	
	6.3 RCV Letdown < 5 m^3^/h?	Go to 6.6
	6.4 Close RCV 227VP	
	6.5 Return	
	6.6 RCV 227VP open?	Go to 6.9
	6.7 Confirm open RCV 048VP and 050VP	
	6.8 Close RCV 227VP	
	6.9 Open three RCV letdown orifices	
	6.10 Regulate RCV charging at compatible flowrate	
	6.11 RCV Charging < 30 m^3^/h?	Go to 6.17
	6.12 Return	
	6.13 Close RCV 002VP and 003VP	
	6.14 Close RCV 007VP, 008VP and 009VP	
	6.15 Close RCV 046VP	
	6.16 Close RCV 050VP	
	6.17 Return	
7. P.RCP STABILIZATION (PZR FULL)	7.1 Regulate the RCPp inj. flowrate to 1.5 m^3^/h/pump	
	7.2 Close RCP 001VP	
	7.3 Close RCP 002VP	
	7.4 Close RCV 227VP	
	7.5 RCV Letdown < 5 m^3^/h?	Go to 7.8
	7.6 Stabilize P.RCP at the reached value by heaters	
	7.7 Return	
	7.8 Open RCV 048VP	
	7.9 Open RCV 050VP	
	7.10 PZR saturated?	Go to 7.20
	7.11 Put two RCV letdown orifices IS	
	7.12 Regulate RCV charging flowrate to minimum compatible	
	7.13 Stabilize P.RCP at the reached value by heaters	
	7.14 RCV Charging < 30 m^3^/h?	Go to 7.16
	7.15 Return	
	7.16 Close RCV 007VP, 008VP and 009VP	
	7.17 Close RCV 046 VP	
	7.18 Close RCV 050VP	
	7.19 Return	
	7.20 Keep only one RCV letdown orifice IS	
	7.21 Put all the heaters IS	
	7.22 Stabilize P.RCP at the reached value by the RCV charging flowrate	
	7.23 Go to 7.14	
8. RCV CHARGING	8.1 At least one RCV charging pump IS?	Go to 8.15
	8.2 Close RCV 046VP	
	8.3 Close RCV 048VP	
	8.4 Close RCV 050VP	
	8.5 RCV 048VP open?	Go to 8.18
	8.6 RCV 050VP open?	Go to 8.20
	8.7 Confirm RCV 227VP closed	
	8.8 RCV 227VP closed?	Go to 8.12
	8.9 Regulate RCV charging flowrate > 6 m^3^/h by RCV 046VP	
	8.10 RCV charging > 6 m^3^/h?	Go to 8.12
	8.11 Return	
	8.12 Close RCV 046VP	
	8.13 Close RCV 050VP	
	8.14 RCV 050VP closed?	Go to 8.16
	8.15 Return	
	8.16 Ask to implement RFLE sheet N° LE 100 (by the locally derived control source breaker)	
	8.17 Go to 8.15	
	8.18 Ask to implement RFLL sheet N° LL143 (Opening RCV 048VP)	
	8.19 Return	
	8.20 Ask to implement RFLL sheet N° LE102 (Opening RCV 050VP by the locally derived control source breaker)	
	8.21 Return	
9. PZR LEVEL CONTROL	9.1 RCV 227VP open?	Go to 9.6
	9.2 RCV Letdown < 5 m^3^/h?	Go to 9.4
	9.3 Return	
	9.4 Confirm one RCV letdown orifice IS	
	9.5 Return	
	9.6 RCV Charging > 6 m^3^/h	Go to 9.5
	9.7 L.PZR < −4 m	Go to 9.13
	9.8 Augment the RCV charging flowrate	
	9.9 Regulate the RCPp inj. flowrate to 3 × 1.5 m^3^/h/pump	
	9.10 RCV Letdown > 5 m^3^/h?	Go to 9.12
	9.11 Regulate the number of RCV letdown orifices IS	
	9.12 Return	
	9.13 L.PZR < 1.35 m	Go to 9.15
	9.14 Go to 9.11	
	9.15 Reduce the RCV charging flowrate	
	9.16 Go to 9.11	
10. TRANSITION TO CHARGING CONFIGURATION	10.1 Close RCV 227VP	
	10.2 Set RCV 046VP at 20%	
	10.3 RCV 046VP closed?	Go to 10.6
	10.4 Ask to implement RFLL sheet N° LL146 (Opening RCV 046VP)	
	10.5 Return	
	10.6 Open RCV 222VP	
	10.7 Open RCV 223VP	
	10.8 RCV 222VP open?	Go to 10.25
	10.9 RCV 223VP open?	Go to 10.25
	10.10 HHSI IS?	Go to 10.23
	10.11 Close RIS 032VP	
	10.12 Close RIS 033VP	
	10.13 RIS 032VP closed?	Go to 10.16
	10.14 RIS 033VP closed?	Go to 10.16
	10.15 Return	
	10.16 Close RIS 034VP	
	10.17 Close RIS 035VP	
	10.18 RIS 034VP closed?	Go to 10.21
	10.19 RIS 035VP closed?	Go to 10.21
	10.20 Go to 10.15	
	10.21 Ask to implement RFLL sheet N° LL160 (Closing BIT isolations)	
	10.22 Return	
	10.23 Ask to implement RFLL sheet N° LL145 (Closing RIS 020VP)	
	10.24 Return	
	10.25 Ask to implement RFLL sheet N° LL139 (Opening miniflow lines of charging pumps)	
	10.26 Return	
11. COMPLETE ISOLATION OF A RADIOACTIVE SG	11.1 Close VVP 001VV by normal way	
	11.2 Set GCT 404KU on EXTERNAL (GCT 131VV)	
	11.3 Set GCT 403KU on AUTO (GCT 131VV)	
	11.4 Open GCT 128VV (GCTa isolating valve)	
	11.5 Close VVP 130VV	
	11.6 Close VVP 127VV (TAFP steam)	
	11.7 VVP 001VV closed?	Go to 11.16
	11.8 Close VVP 140VV	
	11.9 At least one ASG pump IS?	Go to 11.18
	11.10 Reset ASG valves by ASG 001KG and 002KG	
	11.11 Close ASG 012VD	
	11.12 Close ASG 013VD	
	11.13 Close ARE 052VL	
	11.14 Close ARE 054VL	
	11.15 Return	
	11.16 Confirm the steam isolation by action on VVP 001TO and 002TO	
	11.17 Go to 11.8	
	11.18 APG 002KG on BLOCK	
	11.19 Put two ASG MAFP IS	
	11.20 Go to 11.10	
12. COMPLETE ISOLATION OF A SG WITHOUT RADIOACTIVITY	12.1 Reset ASG valves by ASG 001KG and 002KG	
	12.2 Close ASG 012VD	
	12.3 Close ASG 013VD	
	12.4 Put MFP OS	
	12.5 Close ARE 052VL	
	12.6 Close ARE 054VL	
	12.7 Put APG on this SG OS	
	12.8 Close APG 004VL	
	12.9 Close VVP 130VV	
	12.10 Close VVP 001VV using its normal way	
	12.11 Close VVP 127VV (TAFP steam)	
	12.12 Close GCT 131VV by GCT 403KU	
	12.13 Close GCT 128VV (GCTa isolation valve)	
	12.14 VVP 001VV closed?	Go to 12.17
	12.15 Close VVP 140VV	
	12.16 Return	
	12.17 Confirm the steam isolation by VVP 001TO and 002TO	
	12.18 Go to 12.15	
13. ISOLATION OF THE STEAM CONSUMERS	13.1 Set GCT 404KU on EXTERNAL (GCT 131VV)	
	13.2 Set GCT 403KU on AUTO (GCT 131VV)	
	13.3 Close VVP 140VV	
	13.4 Return	
14. PARTIAL ISOLATION OF A SG WITHOUT RADIOACTIVITY	14.1 Close VVP 130VV	
	14.2 Close VVP 001VV using its normal way	
	14.3 Close VVP 127VV (TAFP steam)	
	14.4 Set GCT 404KU on EXTERNAL (GCT 131VV)	
	14.5 Set GCT 403KU on AUTO (GCT 131VV)	
	14.6 VVP 001VV closed?	Go to 14.10
	14.7 Close VVP 140VV	
	14.8 Close APG 004VL	
	14.9 Return	
	14.10 Confirm the steam isolation by action on VVP 001TO and 002TO	
	14.11 Go to 14.7	
15. PUT APG OS	15.1 Set APG 502KU to 0%	
	15.2 Close APG 004VL, 005VL, 006VL and 010VL	
	15.3 Set REN 023KC on CONTAINMENT ISOLATION	
	15.4 Return	
16. PUT AUXILIARY SPRAY	16.1 Regulate RCV charging flowrate to 6 m^3^/h by RCV 046VP on MANU	
	16.2 RCV Charging flowrate < 9 m^3^/h?	Go to 16.14
	16.3 Open RCV 227VP	
	16.4 RCV 227VP open?	Go to 16.13
	16.5 Close RCV 050VP	
	16.6 RCV 050VP closed?	Go to 16.12
	16.7 Put the normal spray OS	
	16.8 RCV letdown < 5 m^3^/h?	Go to 16.10
	16.9 Return	
	16.10 Confirm open one RCV letdown orifice	
	16.11 Return	
	16.12 Ask to implement RFLE sheet N° LE100 (Closing RCV 050VP by the locally derived control source breaker)	
	16.13 Return	
	16.14 Close RCV 227VP	
	16.15 Return	
17. PUT CHARGING IS	17.1 Open RCV 048VP	
	17.2 Open RCV 050VP	
	17.3 RCV 048VP closed?	Go to 17.6
	17.4 RCV 050VP closed?	Go to 17.8
	17.5 Return	
	17.6 Ask to implement RFLL sheet N° LL143 (Opening RCV 048VP)	
	17.7 Return	
	17.8 Ask to implement RFLE sheet N° LE 102 (Opening RCV 050VP by the locally derived control source breaker)	
	17.9 Return	

Note: RCV—chemical and volume control system; IS—in service; OS—out of service; ASG—auxiliary water supply system; TAFP—turbine-driven auxiliary feedwater pump; SG—steam generator; GCT—generator-controlled temperature; PZR—pressurizer; RCP—reactor coolant pump; P.RCP—primary coolant pump; MAFP—motor-driven auxiliary feedwater pump; VVP—vapor from vessel to protection; APG—atmospheric steam dump system.

## Appendix B. Full Eye-Tracking Feature Dictionary

Appendix B provides the complete variable dictionary after Table 1 was compressed for readability in the main text.

**Table A2:** Complete eye-tracking variables used in the PLSR model.

Category	Feature	Description
Time baseline	duration_s	Valid recording duration for each participant during each procedural task.
Fixation	fixation_count	Number of detected fixations during the task; reflects the frequency of visual sampling.
Fixation	fixation_rate_per_min	Number of fixations per minute, used to reduce the influence of task duration.
Fixation	fixation_mean_duration_ms	Mean fixation duration, related to visual dwell time and information-processing demand.
Fixation	fixation_median_duration_ms	Median fixation duration, less affected by extreme fixation values.
Fixation	fixation_sd_duration_ms	Standard deviation of fixation duration; reflects instability in fixation rhythm.
Fixation	fixation_total_prop	Proportion of total recording time spent in fixation.
Saccade	saccade_count	Number of detected saccades during the task; reflects gaze-shift frequency.
Saccade	saccade_rate_per_min	Number of saccades per minute.
Saccade	saccade_mean_duration_ms	Mean saccade duration.
Saccade	saccade_median_duration_ms	Median saccade duration.
Saccade	saccade_sd_duration_ms	Standard deviation of saccade duration.
Saccade	saccade_total_prop	Proportion of total recording time spent in saccades.
Pupil	pupil_mean_mm	Mean pupil diameter, indicating overall physiological arousal during the task.
Pupil	pupil_sd_mm	Standard deviation of pupil diameter, indicating pupil fluctuation.
Pupil	pupil_cv	Coefficient of variation of pupil diameter, calculated as the standard deviation relative to the mean.
Pupil	pupil_p95_mm	95th percentile of pupil diameter, reflecting upper-end dilation during demanding segments.
Spatial distribution	gaze_x_sd_px	Standard deviation of horizontal gaze coordinates.
Spatial distribution	gaze_y_sd_px	Standard deviation of vertical gaze coordinates.
Spatial distribution	gaze_dispersion_px	Two-dimensional gaze dispersion.
Gaze dynamics	gaze_path_per_s	Gaze path length per second.
Gaze dynamics	gaze_speed_mean_px_s	Mean gaze movement speed.
Gaze dynamics	gaze_speed_sd_px_s	Standard deviation of gaze movement speed.
Spatial distribution	spatial_entropy_4×3	Spatial entropy of fixations based on a 4 × 3 screen grid; reflects the dispersion of visual search.
Fixation transitions	fixation_transition_mean_px	Mean transition distance between adjacent fixation points.
